# Non-standard issues in business finance: an overview

**DOI:** 10.1007/s11573-022-01122-8

**Published:** 2022-11-17

**Authors:** Wolfgang Breuer, Andreas Pfingsten

**Affiliations:** 1 Department of Finance, RWTH Aachen University, Aachen, Germany; 2 Finance Center Münster, WWU Münster, Münster, Germany

**Keywords:** G00, G3, G32

## Introduction

This special issue is about “non-standard issues in business finance”. Apparently, in order to decide which papers are eligible for this special issue it is necessary to define some kind of “standard” in business finance as a benchmark, so that any deviation from this standard qualifies for this issue. Alas, when singling out some “standard”, this certainly is not independent of time and place. Taking payments as a simple example from another area, cash payments have lost market share over the last decade but are still dominating in Germany (Deutsche Bundesbank 2022), whereas in the U.S. with its ample evidence of the relevance of credit card payments, several cities and states have deemed it necessary to establish regulations requiring retail stores to accept cash (Tarlin 2021, p. 2).

There are various dimensions according to which one may distinguish between a standard-case and a non-standard one in business financing. First of all, “standard” may be defined according to the financial instruments under consideration with (common) equity (i.e. issuing shares) and debt (i.e. issuing bonds or borrowing from a bank) traditionally being regarded as standard while all other kinds of financial instruments, in particular the *“hybrid” mezzanine instruments*, are classified as “non-standard issues”. In a similar way, the analysis of the whole domain of *supporting legal or contractual rights* that stabilize financial decisions may give rise to non-standard issues.

Alternatively, cultural differences, frequently related to geographical origins, may provide benchmarks. As a rather rough classification one may simply single out the field of *international finance*, implicitly stating standard issues to have always a more or less domestic orientation. For example, German scholars would typically subscribe to a secular view of business financing as being standard, implying that religious aspects as incorporated in studies on *Islamic finance* are “non-standard”. A third categorization would rely on the objective function underlying financial decision-making in firms. Simple shareholder value maximization may then be regarded as standard and a broader stakeholder orientation, e.g., in order to account for sustainability goals, refers once again to a non-standard case, thus defining the field of *sustainable finance*. In addition, as another example of the temporal dimension with standard issues describing on the one hand business financing in the recent past or the present in contrast to current developments reaching out for the future, we may refer to what is coined *digital finance*. For some it is still non-standard today, but may become a standard component of business financing in the not too distant future. In contrast to other categories, digital finance exhibits two distinct dimensions. On the one hand, this term refers to the increasing relevance of digitalization in financial decision making, e.g., when applying methods of artificial intelligence for credit scoring. On the other hand, utilizing such methods for empirical analyses of finance related issues by scholars is also referred to as “digital finance”. As still another classification, one may distinguish between “normal times”, whatever that may mean, and “times of crises” leading to non-standard “*corporate finance in times of crises*”, e.g. in the aftermath of the financial crisis 2008/09 or the COVID-19 pandemic. Certainly, there are even more ways in which to differentiate between standard and non-standard issues in business finance.

As already pointed out, the understanding of “standard issues” may vary over time. In order to get a feeling for recent developments in the field of finance, we present in the following Sect. 2 an overview of the relevance of various kinds of topics in corporate finance at the annual meetings of the European Finance Association (EFA), the most prestigious European finance conference.^1^ Section 3 then introduces the papers of this special issue and puts them into perspective to our findings of Sect. 2. Section 4 concludes.

## Corporate finance as a major subject at EFA conferences

According to our introduction, we look at annual meetings of the European Finance Conference from 2009 to 2021. In a first step, we select all papers that are subsumed under headings related to “Corporate Finance”. In most years, this refers to the two main categories “Corporate Finance, Theoretical” and “Corporate Finance, Empirical” (in 2016 it was Corporate Finance and Governance, Theoretical/Empirical). However, in the years 2014 and 2015 the organizers of the annual meetings refrained from defining encompassing larger categories. Instead, here we are left only with the headings for all of the about 70 sessions per meeting. We thus had to decide manually whether a session like “Distress and Renegotiation” or “Ethics Meets Finance” should be labelled “related to corporate finance” or not. Certainly, in many cases like “Behavioral Corporate Finance” or “Financial Policy: Theory” this was not too difficult a task. Nevertheless, when in doubt, we decided to take the respective papers into account. As Table 1 reveals, in 2014 and 2015 we thus reached a peak in the share of papers with a corporate finance context in relation to all papers of the corresponding annual meeting.

**Table 1:** Overview of total number of papers under consideration

Year	2009	2010	2011	2012	2013	2014	2015	2016	2017	2018	2019	2020	2021
(1) # Examined papers	51	50	54	37	23	88	74	62	37	49	65	66	48
(2) # Missing corresponding documents	0	1	0	2	1	6	7	4	1	2	4	0	0
(3) Total number of corporate finance papers	51	51	54	39	24	94	81	66	38	51	69	66	48
(4) = (1)/(3)	100.00%	98.04%	100.00%	94.87%	95.83%	93.62%	91.36%	93.94%	97.37%	96.08%	95.55%	100%	100.00%
(5) Total number of conference papers	215	217	201	216	240	267	240	243	222	243	243	243	180
(6) = (3)/(5)	23.72%	23.50%	26.87%	18.06%	10.00%	35.21%	33.75%	27.16%	17.12%	20.99%	28.40%	27.16%	26.67%

For all these 732 papers from the years 2009 to 2021, we tried to retrieve the whole manuscripts. Also according to Table 1, our “success rate” lies in the interval of 90% to 100%. For the 704 papers which we were able to retrieve, we determined whether they make use of words from specific lists which we utilize to define several different thematical categories. Category #1 is called by us “classical corporate finance”, Category #2 “hybrid finance”, Category #3 “supporting legal or contractual rights”, Category #4 “international finance”, Category #5 “corporate finance in times of crises”, Category #6 “Islamic finance”, Category #7 “sustainable finance”, and Category #8 “digital finance”. The respective word lists are described in detail in Table 2.

**Table 2:** Word lists for identifying different thematical categories of corporate finance

Category	Word list
#1: Classical corporate finance	Debt, equity, capital structure, leverage, trade-off theory, pecking order, market timing, agency, internal financing, external financing, bankruptcy, loan, tradeoff, stock, bond
#2: Hybrid finance	Mezzanine, leasing, hybrid, derivative, option, convertible, floating rate note, financial innovation
#3: Supporting legal or contractual rights	Maturity, seniority, collateral, control rights, financial intermediation, bank financing
#4: International finance	Foreign currency, exchange rate, foreign exchange, hedg*, speculat*
#5: Corporate finance in times of crises	Covid-19, crisis, crises, bubble, crash, collaps*, disaster, systemic, pandemic
#6: Islamic finance	Islam, shari´ah, sukuk, shariah, haram, harām, fiqh, sunna, ribā, riba, gharar, maysir, qimār, qimar, koran, wadīʿa, wadiah, hibah, wadiah yad amanah, wadiah yad dhamanah, mudaraba, qard al-hassan, qard hassan, marabaha, tawarruq, istina, idschara, muscharaka
#7: Sustainable finance	Sustainab*, gender, divers*, equality, pollution, social, carbon, climate, ecolog*, environment*, esg, csr, impact invest*, female, race, racial, discriminat*, woman, women
#8: Digital finance	Text analysis, artificial intelligence, machine learning, digital finance, fintech*, insurtech*, neural, network, forest regression, word list

**Fig. 1:**
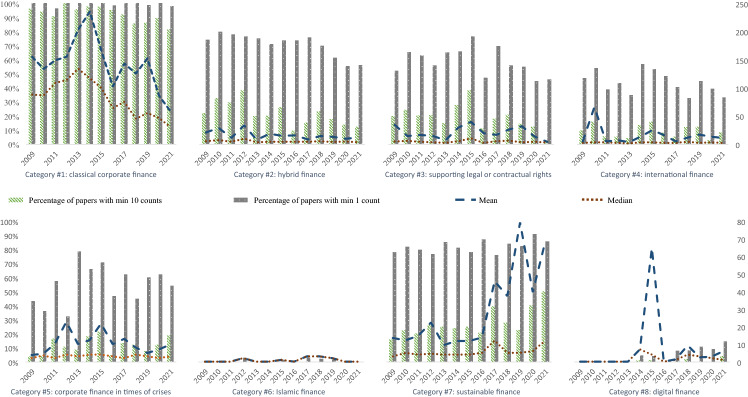
Relative Importance of Categories #1 to #8 at EFA Conferences 2009 to 2021

For each paper we examined how often words from the respective word list are used. Based on these word counts we determine four main figures: First of all, we compute the share of all corporate finance papers that refer to a specific category in a specific year. In addition, we also identify the fraction of those papers with at least ten occurrences of words from a specific word list (multiple appearances of the same word are allowed). Third, the average number of words referring to a certain list based on all papers with a respective reference is determined. Fourth, the corresponding median value is depicted. All findings are summarized in Fig. 1. In order to make results easy to read, the scale on the right-hand side for the mean and median of absolute word counts differs between the diagrams for Categories #1 to #4 and those for Categories #5 to #8.

On average, about 25% of all presentations of a typical annual meeting are devoted to corporate finance issues. In what follows, we call these the “corporate finance papers”. And of these corporate finance papers, as expected, a considerable share exhibits words related to *classical* corporate finance issues. Apparently, our manual and thus somewhat arbitrary selection of corporate finance papers for the years 2014 to 2016 does not seem to be too inadequate, as according to Category #1 of Fig. 1 the share of corporate finance papers for 2014 and 2015 neatly fits into the overall picture. Nevertheless, in this special issue we are more concerned with non-standard issues in business financing.

One of the most striking results is the increase in relative importance of papers in the domain of digital finance (see Category #8 in Fig. 1). While there are no papers of this category before 2014, the fraction increases to as much as 14% in 2021. There is some similar positive, though less pronounced surge in the relevance of papers from the sustainable finance category—at least if we focus on the share of papers with a minimum of ten topic related word counts (see Category #7 in Fig. 1). In contrast to these topics with a recent positive trend, corporate finance papers related to crisis topics do not exhibit a clear focus in the aftermath of 2009 or 2020, though there seems to be some peak in the years 2013 to 2015. Maybe discussing crisis related issues is a long term task and hence there is some time lag on the one side and maybe on the other side, the rise in interest is permanent, obscuring the identification of clear temporal foci (see Category #5 in Fig. 1). Another clear finding is that issues of Islamic finance are of almost no importance and hence certainly only constitute a thematical niche—at least at the annual meetings of the European Finance Association (see Category #6 in Fig. 1). More “established” non-standard issues in corporate finance like “hybrid finance” (Category #2 in Fig. 1) and the investigation of supporting legal and contractual rights (Category #3 in Fig. 1) are indeed of relevance. However, in recent years, their relative importance has been reduced—maybe as a consequence of an increasing relevance of categories like digital finance and sustainable finance. Last, not least, something similar seems to hold for international finance, though on a generally somewhat lower level. (Category #4 in Fig. 1).

Summarizing, the established fields of international finance, of hybrid finance, and of the analysis of supporting legal and contractual rights indeed play a major, though somewhat declining role in the realm of non-standard issues in business financing, while topics like digital finance and sustainable finance are—not too surprisingly—on the rise. At least since the early 2010s, corporate finance in times of crises seems to be established as well, while Islamic finance still is of almost no importance. Against this background, we can now turn to the presentation of the papers of this special issue.

## The papers of this special issue

Debt and equity are the core types of business financing. They come as plain vanilla versions of loans, bonds, and common stock, but also in more sophisticated versions. In many cases, features of equity and debt are combined to create hybrid forms, often referred to as mezzanine capital. Business financing in practice goes far beyond the choice between equity and debt, which is irrelevant under ideal market conditions according to the seminal paper of Modigliani and Miller (1958), and aims at optimizing the mix of loans, bonds, common stock, and many other feasible facilities.

The adequacy of a certain way of financing a business differs depending on the particular situation of the firm and the economy. Young firms in particular will typically not be able to obtain loans or issue debt.^2^ Information asymmetries as well as interest payments insufficient to compensate financiers for the relatively high probability of losses are among the reasons. Therefore, young firms will have to rely more on equity financing, which sometimes comes with the additional advantage of investors who support the management with their own experience and networks.

The first paper, “Why deep pockets make great borrowers—An empirical analysis of venture loans,” by Nico Lehnertz, Carolin Plagmann, and Eva Lutz, obviously belongs to Category #1, classical corporate finance (Lehnertz et al. 2022). It classifies for this issue by investigating a particular case of venture debt, namely venture loans, an under-researched hybrid instrument suitable for the financing of young firms (Category #2, hybrid finance). Venture loans consist of two components, a classic loan and a warrant. They are typically provided by specialized funds. Although the market share of venture loans is small, they are still sizable in absolute terms. In some sense the involvement of an institutional investor substitutes collateral as the typical protection against all types of uncertainties. In order to assess the relevance of the instrument, it is important to know the availability of venture loans. The interaction of investors and firms indicates that an investigation should look at start-ups as well as investors.

The authors formulate a number of hypotheses covering features of both groups. A first and arguably unsurprising finding is that more mature start-ups are more likely to obtain venture loans. Maturity in this analysis is not measured in terms of age and years, but as the number of the financing round in which venture loans are obtained. Therefore, maturity in this setting can be seen as an indicator of reputation because a higher number of the financing round means that the firm has demonstrated before that it develops along the lines expected by earlier investors. In addition, higher financing rounds are closer to the likely profitable exit. More surprising is the second observation. The number of patents the firm holds is not positively related to the probability that it will obtain a venture loan. Investors may be concerned that patents held by young firms are relatively specific and non-marketable. Third, regarding the start-up’s industry, it turns out that firms from the medical, health, and life science industries are more likely to obtain venture loans, but firms from the semiconductor and biotechnology industries are not. When recognizing the latter results, one should keep in mind that the data end in 2020, and hence positive effects of the pandemic, which may have improved the perception of biotechs, are not yet included.

Turning now to the investors, the authors find that investors’ commitment, measured in terms of average capital per investor, has a statistically significant positive impact on the probability of receiving a venture loan. A higher financial commitment in terms of having more skin in the game apparently comes along with a higher involvement of the investor and is likely combined with less information asymmetry. The results also indicate that investors with predominantly financial interests are more likely to provide venture loans than investors with strategic objectives.

Firms permanently need to obtain additional financial means. According to Modigliani and Miller (1958), the capital structure is irrelevant for firm value and cost of capital under the assumption of a perfect capital market in equilibrium. In this context, a “perfect” capital market is nothing more than a benchmark model featuring some simplifying assumptions which mainly aim at rendering rigorous analyses easier. It is by no means an unconditional synonym for a “desirable” capital market. Anyway, by now we are aware of a number of real-life deviations from the simplifying picture of the perfect capital market underlying the irrelevance theorem, and hence the choice between equity and debt really matters. The pecking order theory by Myers and Majluf (1984) offers reasons for the sequence in which different types of financing should be chosen and helps to explain why in particular situations a certain facility is selected. The classical survey by Harris and Raviv (1991) examines the pecking order theory as well as other models concerning firm capital structure and related empirical evidence. In contrast to the pecking-order theory, the trade-off theory compares positive and negative consequences of different financing alternatives (e.g., regarding the exploitation of tax benefits or the avoidance of bankruptcy costs) in order to determine an overall optimal capital structure for a firm. Hackbarth et al. (2007) demonstrate that the trade-off theory can explain many stylized facts. López-Gracia and Sogorb-Mira (2008) investigate the financing of small and medium-sized enterprises (SMEs) and are, therefore, closely related to the paper by Lehnertz et al. (2022). They show that both the trade-off and the pecking-order theory have some virtue in explaining a firm’s capital structure.

In such empirical studies, at best, only realized transactions are publicly observable. Private transactions may be hidden completely. And so are intended transactions. This is where the paper “Nothing but Good Intentions—The Search for Equity and Stock Price Crash Risk” by Doron Reichmann, Rouven Möller, and Tobias Hertel starts (Reichmann et al. 2022).

It is known that managers tend to hoard bad information about the firm, instead of publishing it, in order to avoid stock price crashes coming along with losses in their own reputation and compensation. This paper addresses another reason why managers might hide bad information from the public. If they intend to raise equity for whatever reason, any negative news may reduce the volume of equity that can be raised or decrease its price. An actual increase in equity can be observed, but it is typically not known, whether or not managers would have liked to increase equity but have refrained from it due to adverse conditions. Thus, in the first step, the authors construct a continuous measure of managers’ intentions to raise equity. They do so by evaluating 10-K files.

It turns out that equity intent, i.e. the intention to raise equity, is positively related to the risk that the stock price will crash. In fact, this matches quite well the theoretical background of the pecking-order theory as the latter is based on the observation that managers have an incentive to issue equity in times of overvaluation. Apparently, individual overvaluation is most pronounced in times of looming (idiosyncratic) crises. The message to investors is that they should be careful when planning to invest into firms with large equity intent according to the text of their 10-K files, because this intention will expose them to above average risk for a stock price crash. The same message is known from the pecking-order theory: as investors should generally be quite skeptical about the prospects of firms issuing equity, Myers and Majluf (1984) recommend using equity financing only as a means of last resort to avoid negative stock market reactions triggered by suspicious investors.

The authors then continue by showing that hoarding of bad news is the mechanism that pulls investors towards supplying firms with equity that are exposed to higher crash risks. Two ways of bad news hoarding are investigated in more detail. One is earnings manipulation, the other textual obfuscation. Essentially, the classical agency problem of bad news hoarding is investigated with more sophisticated textual analyses showing that the intention to raise equity can be recovered from 10-K files. In particular, bad news hoarding through textual obfuscation seems to be important yet under-researched. While the content of the paper is deeply rooted in Category #1, classical corporate finance, at the same time, from a methodological point of view, this paper is also a representative of Category #8, digital finance, an area that appears to gain quickly in importance as outlined in the previous section.

Whether firms succeed in raising the desired equity amount depends on firm data. But data on the real economy and on the financial sector certainly matter, too, and so may the political situation or a general sentiment. The COVID-19 pandemic is a particular case. The real economy was hit first of all, the financial sector was infected with a delay. Of course, some industries are being hit more than others, and some even may benefit from the crisis. At any rate, the typical outcome is a worse economic situation than before and a negative sentiment of investors. In their paper “Tough times for Seasoned Equity Offerings: Performance during the COVID pandemic,” Mark Zenzius, Christian Flore, and Dirk Schiereck look, based on US data, at the wealth effect of SEO announcements (Zenzius et al. 2022).

Apart from the results as such, the really interesting question is how effects during a crisis differ from those for periods with no crisis. Indeed, this may be considered the core question of Category #5 of the preceding section, corporate finance in times of crises. Therefore, the authors start by comparing various economic indicators. In order to increase the general relevance of their study, they also compare the COVID-19 pandemic to the likewise global financial crisis 15 years ago. Among others, it turns out that the downturn was much faster and larger during the pandemic, and similarly the upswing was faster and larger, too. Concerning the volume of seasoned equity offerings (SEOs), there is rather a small effect, if any, during the financial crisis, but a large increase in 2020. It is well established, theoretically and empirically, that seasoned equity offerings are usually a negative signal and lead to negative price reactions (due to the overvaluation problem described above).^3^ This is the case in good times and it should not be surprising to see a similar effect during the COVID-19 pandemic. The authors hypothesize that the effect is even more negative during the COVID-19 pandemic. A potential reason might be that problems of informational asymmetry get worse in times of crises due a higher level of uncertainty regarding future prospects. However, even in a global crisis there exist winners. On the aggregate level, it is plausible that some industries gain while the others lose. In these winner industries, we may not see the negative price impact of an SEO announcement, because investors may find it reasonable that these winner firms need additional capital for expansion in order to exploit a situation which is beneficial for them. The authors therefore compare a number of different industries.

In an event study, the authors find a highly significant negative price impact on the day of the announcement. In line with this result, the cumulative abnormal average returns for different event windows are also significantly negative. Comparing the numbers with those reported in a meta analysis of SEO announcement studies, the numbers during the COVID-19 pandemic are clearly larger. This is empirical support for the hypothesis that the negative effects of SEO announcements are larger during a crisis. A cross-sectional analysis of drivers of this effect reveals that among the number of variables more or less only the size of the firm, measured as market capitalization, has a statistically significant effect. Larger firms experience less negative effects when announcing an SEO. Smaller negative effects are also seen for firms from the presumably COVID-19 related biotech and healthcare industries, which seems plausible. However, if these firms have lower price-to-book values, then the announcement effect is more negative. It seems that the market does not believe that investments financed by new equity will turn such firms into winners.

Stock prices react to capital measures and to changes in the general economic situation, as in the paper just described, but they may also react when governments introduce fiscal, monetary, or any other measures to counteract the effects of a crisis. Banks as financial intermediaries are hit in a pandemic only later when they begin to feel the damage firms are exposed to. However, banks come in different types and with different business models, and the obvious question is whether different types of banks are affected differently by governmental actions. When comparing different types of banks, it is important that they are as similar as possible with respect to all features except the one which is to be compared. In particular, it is desirable that these banks are active in the same country.

In a similar way as the irrelevance theorem for firms’ financing decisions, the theory of financial intermediation tells us that there is no rationale for banks and other kinds of financial intermediaries when capital markets are perfect (Diamond 1984; Freixas and Rochet 2008). However, under imperfect market conditions there even may be room for different kinds of banks at the same time. Dual banking systems with conventional banks and Islamic banks operating simultaneously are the research field of Amal Alabbad and Andrea Schertler, obviously belonging to Category #6, Islamic finance (Alabbad and Schertler 2022), and being analyzed only relatively rarely according to Fig. 1 above. In their paper “COVID-19 and bank performance in dual banking countries: An empirical analysis,” the authors examine the impact of workplace closures on the one hand and income support schemes as well as debt relief programs on the other on the performance of conventional banks and Islamic banks, respectively. Islamic banking, as a short version for describing banks that adhere to the principles of Sharia, differs in a number of aspects from conventional banking. Arguably most importantly, Islamic banks tend to avoid interest-bearing transactions, but instead use other modes including some kind of profit and loss sharing or asset-based finance. Here, the predominant market “imperfection” is the existence of utility not driven solely by financial considerations but by the desire to cling to certain religious tenets. It is therefore conceivable, that, first, the two types of banks have different customer bases, and, second, they are affected differently by economic changes in general and by government interventions in particular.

The authors present several findings. First, there is a strong co-movement of incomes of Islamic banks and conventional banks during the COVID-19 pandemic. The finance/interest income of Islamic banks and the interest income of conventional banks both decrease, whereas the non-finance, respectively non-interest, incomes increase in some quarters. The latter also holds for net income. There are hardly any differences between Islamic banks and comparable conventional banks in the matched sample. Income support schemes, which facilitate it for debtors to fulfill their financial obligations, may theoretically improve banks’ income. Yet the empirical analysis shows that they have almost no effect on conventional banks’ net income, but they are favorable for Islamic banks’ net income. For debt relief programs, there is no clear picture, possibly because they are highly correlated with workplace closure recommendations. A search for possible reasons reveals that profit and loss sharing contracts in Islamic banks do not seem to be an important factor as they account for only a relatively small share of income. Turning from income changes to stock-price responses, the more beneficial effect for Islamic banks prevails. It is difficult to provide definite reasons for these observations. It seems quite likely, however, that the stronger focus of Islamic banks on private customers is a major factor because these clients are more likely to repay the debt when income support programs are introduced.

All of the studies presented so far have one feature in common. Firms obtain liquidity either from financial intermediaries, e.g. specialized funds in the case of venture loans, or from investors directly as, for example, in the case of SEOs. The final paper “The ‘C’ in crowdfunding is for co-financing—Exploring participative co-financing, a complement of novel and traditional bank financing,” by Carolin Bock, Sven Philipp Siebeneicher, and Jens Rockel deviates from this setting (Bock et al. 2022). They combine crowdfunding, i.e. collecting liquidity from individual investors via a platform, with traditional bank funding^4^ and as such the paper belongs to Category #8, digital finance, because online platforms require extensive digitalization.

The authors’ basic claim is that crowdfunding and regional banks fit together quite well. Having particularly German savings banks in mind, the financing of entrepreneurs in start-ups and SMEs is an essential task for these banks, but also leaves room for participation of other financiers. This holds in the area of debt financing, which turns out to be preferred, but also in equity financing. As their theoretical foundation the authors refer to social capital theory and the technology acceptance model. Their survey-based analysis reveals plausible intentions of decision makers in regional banks, most prominently the idea to reap rents from cross-selling and to be perceived as innovative. Measuring perceived usefulness is discussed in detail. Factors that matter for the relations analyzed are the ease of use of the instrument, measured by know-how, and the prior experience with crowdfunding as such.

## Outlook

In total, the papers in this issue address very different questions. They all have in common contributions to business financing that in one aspect or the other deviate from mainstream analyses. Therefore, they may also serve as an encouragement to leave beaten tracks when choosing research topics.

Looking again at Fig. 1, it seems plausible that Categories #7, sustainable finance, and #8, digital finance, will grow in importance. With respect to *sustainable finance*, not least regulatory requirements and public awareness will continue to put pressure on firms, financial and non-financial, to take sustainability into account when it comes to business models and financing decisions. This will open a wide range of research questions in finance.

The continued rise of research qualifiable as *digital finance* will likely arise from several sources. Similar to sustainable finance, digital finance will be at the heart of business models, be it in the form of (new) competitors like fintechs, insurtechs, or bigtechs, to name but a few, or in the form of adapted services, e.g., digital currencies, cryptoassets, smart contracts, and many more. Moreover, the methods used for analyzing business finance and other financial topics will be enriched by digital tools, for instance textual analysis as in Reichmann, Möller, and Hertel (2022).

The future development of Islamic finance, Category #6, is much more difficult to predict. On the one hand, according to several internet sources close to 2 billion people (roughly one fourth of the world’s population) adhere to the Islam, making it a highly relevant object for financial research. On the other hand, as some principles and products of Islamic finance require deep institutional knowledge to appreciate their distinctiveness and as they diverge from basic assumptions prevailing in leading journals, successful publication of studies may be more at risk than with mainstream approaches. Unfortunately, this may deter researchers from diving into this field.

What will happen with Category #4, international finance, is another open question. One may argue that its importance will increase due to much more interconnected economies, even if presently some tendencies towards partial autarky are being observed. However, this might imply that eventually finance and international finance become synonyms and the category becomes void. Alas, it is also conceivable that local institutional specifics of financial and non-financial sectors will survive, and much room for international corporate finance papers will remain.

Finally, we would welcome a lack of need for studies on corporate finance in times of crises, Category #5. Regrettably, given the present political situation we are less optimistic than ever that this wish will come ﻿true.

## Footnotes

1 Though somewhat imprecise, we do not distinguish between business finance and corporate finance.

2 Hochberg et al. (2018) point out that technology start-ups are not well suited for obtaining outside debt. Yet the authors also acknowledge a considerable market for it, e.g., in the Silicon Valley.

3 Masulis and Korwar (1986) is a classical paper on this issue, comparing predictions of several theoretical models to empirical facts.

4 Notice that the platform, respectively its operator, can be seen as a financial intermediary.
